# Nonlinear Optical Microscopy for Histology of Fresh Normal and Cancerous Pancreatic Tissues

**DOI:** 10.1371/journal.pone.0037962

**Published:** 2012-05-24

**Authors:** Wenyan Hu, Gang Zhao, Chunyou Wang, Jungang Zhang, Ling Fu

**Affiliations:** 1 Britton Chance Center for Biomedical Photonics, Wuhan National Laboratory for Optoelectronics, Huazhong University of Science and Technology, Wuhan, People's Republic of China; 2 Key Laboratory of Biomedical Photonics of Ministry of Education, Huazhong University of Science and Technology, Wuhan, China; 3 Pancreatic Surgery Center, Union Hospital, Huazhong University of Science and Technology, Wuhan, People's Republic of China; Tufts University, United States of America

## Abstract

**Background:**

Pancreatic cancer is a lethal disease with a 5-year survival rate of only 1–5%. The acceleration of intraoperative histological examination would be beneficial for better management of pancreatic cancer, suggesting an improved survival. Nonlinear optical methods based on two-photon excited fluorescence (TPEF) and second harmonic generation (SHG) of intrinsic optical biomarkers show the ability to visualize the morphology of fresh tissues associated with histology, which is promising for real-time intraoperative evaluation of pancreatic cancer.

**Methodology/Principal Findings:**

In order to investigate whether the nonlinear optical imaging methods have the ability to characterize pancreatic histology at cellular resolution, we studied different types of pancreatic tissues by using label-free TPEF and SHG. Compared with other routine methods for the preparation of specimens, fresh tissues without processing were found to be most suitable for nonlinear optical imaging of pancreatic tissues. The detailed morphology of the normal rat pancreas was observed and related with the standard histological images. Comparatively speaking, the preliminary images of a small number of chemical-induced pancreatic cancer tissues showed visible neoplastic differences in the morphology of cells and extracellular matrix. The subcutaneous pancreatic tumor xenografts were further observed using the nonlinear optical microscopy, showing that most cells are leucocytes at 5 days after implantation, the tumor cells begin to proliferate at 10 days after implantation, and the extracellular collagen fibers become disordered as the xenografts grow.

**Conclusions/Significance:**

In this study, nonlinear optical imaging was used to characterize the morphological details of fresh pancreatic tissues for the first time. We demonstrate that it is possible to provide real-time histological evaluation of pancreatic cancer by the nonlinear optical methods, which present an opportunity for the characterization of the progress of spontaneous pancreatic cancer and further application in a non-invasive manner.

## Introduction

Pancreatic cancer is the fourth leading cause of cancer-related mortality in the worldwide, with an overall 5-year survival rate of 1–5% [1]. Better treatment can contribute to a significant improvement in patient survival [2]. Intraoperative consultation which mainly involves the examination of the surgical excised specimen, is important for the surgeon to determine the most appropriate treatment options [3]. However, it is subject to the time constraints. A single frozen section diagnosis with high level of accuracy takes 20 minutes, and it is much longer when multiple frozen sections are required to perform on a single specimen [3]. Real-time histology of fresh or live tissues without sectioning or additional processing, would not only facilitate immediate establishment or confirmation of a diagnosis and stage intraoperatively that will influence the surgical procedure, but also make it possible to evaluate all the surgical margins so that the tumor is removed completely without compromising the normal part of the pancreas. Accurate surgical margin assessment allows the improvement of long-term survival, since positive surgical margins occur among 37–50% of patients undergoing surgical resection and the overall survival of these patients ranges between 8 and 14 months [4]. Real-time detection of morphological patterns at the resolution of a single cell, which is an analogue of histology, indicates an attractive prospect for optimal intraoperative management of pancreatic cancer with favorable survival benefit.

Optical methods, taking advantage of non-invasion and high tempo-spatial resolution, can achieve *in vivo* imaging and sensing in biomedical studies. Raman spectroscopy, which is based on the difference in the energy of the incident and scattered photons due to the molecular vibrations, is sensitive to the changes of chemical composition in cells and tissues. It has been applied to the differentiation of normal and cancerous pancreatic tissues from a mouse model [5]. Reflectance and fluorescence spectroscopy can provide biochemical information of the tissues to distinguish different human pancreatic tissues, including normal pancreatic tissue, pancreatitis, and pancreatic adenocarcinoma [6], [7]. Photon-tissue interaction models have been further developed to provide quantitative links between the reflectance and fluorescence measurements and histological characteristics of human pancreatic tissues, such as the nuclear size [8], [9]. However, the spectral parameters are difficult to be directly matched with the morphological features revealed by the conventional histological examination, especially the changes of nuclear shape and organization of the extracellular matrix. More detailed characterization of the pancreatic morphology with cellular resolution using optical methods is necessary to improve the detection of pancreatic neoplasia, implicating a new means of real-time histology.

In recent years, nonlinear optical microscopy (NOM), primarily including TPEF and SHG, has emerged as a powerful tool to identify slight structural and functional changes at cellular resolution. NOM has the advantage of submicron spatial resolution, millisecond temporal resolution, and the optical sectioning ability in turbid tissues [10], [11]. One important nature of such an imaging modality is that the endogenous optical biomarkers in tissues can be employed to provide contrast, which makes it possible to detect human diseases without the need for fixation, sectioning, or staining. Intrinsic two-photon excited fluorescence (TPEF) biomarkers including reduced nicotinamide adenine dinucleotide (phosphate) [NAD(P)H] and flavin adenine dinucleotide (FAD) have been applied to reveal the morphology of the cells, since NAD(P)H and FAD are the major fluorophores in the cytoplasm [12], [13]. Meanwhile, the collagen fibers, which are important structural proteins in the extracellular matrix (ECM), can implement the intrinsic second harmonic generation (SHG) process in biological tissues to reflect the ECM pattern [12], [13]. In addition, the intracellular NAD(P)H and FAD are also related with the redox ratio of cells, which can be used as an indicator of the metabolic level of cells [14], [15]. NOM has been widely applied to visualize cellular and tissue structures in different cancer tissues including ovarian, bladder, gastric tissues, and so on [14], [16]–[20].

We first, to our knowledge, characterized the morphological details of pancreatic tissues using label-free TPEF and SHG techniques. In an effort to evaluate the feasibility of NOM for the detailed morphological characterization of pancreatic tissues, we applied label-free TPEF and SHG techniques to normal rat pancreas and related with traditional histological staining images. Various routine means for the preparation of the specimens have been compared to acquire optimum nonlinear optical imaging of pancreatic tissues. The chemical-induced pancreatic cancer tissues were further characterized and compared with normal pancreatic specimens to validate the ability of NOM to reveal the neoplastic changes. In order to assess the potential of the label-free TPEF and SHG techniques to characterize different stages during the growth of pancreatic cancer, the subcutaneous pancreatic tumor xenografts harvested at different time points after implantation were quantitatively analyzed based on their morphological characteristics.

## Materials and Methods

### Animal models

A chemical-induced pancreatic cancer model that spontaneously develops malignant cancer in the pancreas was used for comparison of neoplastic tissues to normal tissues. The experiments were approved by the Ethic Committee of Tongji Medical College, Huazhong University of Science and Technology. Animal care was provided in accordance with the procedures outlined in the “Guide for Care and Use of Laboratory Animals” published by the United States National Institutes of Health. The male Sprague-Dawley rats (100–125 g) were obtained from the Experimental Animal Center of Tongji Medical College (Wuhan, China). The tumors were induced according to a previously established protocol [21]. In brief, anesthesia was induced with vaporized ether, followed 10 min later by an intramuscular injection of pentobarbital (20 mg/kg) and ketamine (50 mg/kg). The rats underwent a midline laparotomy with exposure of the pancreatic head segment. The parenchyma was incised parallel to the course of the common bile duct, and a pocket was developed in the pancreatic parenchyma at the incision site. 5 mg of DMBA crystals were implanted and secured in place by means of a 6-0 prolene purse-string suture. The tumors were harvested and imaged at 9 months after induction. Freshly excised tissues from a total of 12 (10 normal and 2 chemical-induced cancer) rats were imaged and compared.

Athymic (nu/nu) mice on a BALB/c background, purchased from Shanghai Laboratory Animal Center (Shanghai SLAC Laboratory Animal Co. Ltd), were used for the establishment of subcutaneous pancreatic tumor model. The experiments followed the procedures approved by the Institutional Animal Ethics Committee of Huazhong University of Science and Technology. Six- to eight-week-old male mice were raised under specific pathogen-free (SPF) conditions, with the temperature maintained at about 21°C and humidity of 60–70% with artificial light for 12 h. The tumor xenografts were developed from the established PANC-1 cell line (ATCC, Rockville, MD) [22]. About 6×10^6^ PANC-1 cells were inoculated subcutaneously to the left blank and the subcutaneous tumor xenografts were harvested at 5, 10, 20, 30 days after implantation, respectively. A total of 20 mice with tumor xenografts (5 mice for each stage) were used for imaging and quantitative analysis.

### Preparation of the pancreatic specimens

The animals were euthanized with an overdose of isoflurane, and then the pancreatic tissues were dissected out and imaged. Before imaging, the freshly excised tissues including the normal rat pancreas, the chemical-induced pancreatic cancer tissues and the subcutaneous tumor xenografts, were incubated in phosphate buffered saline (PBS). Half of each specimen was then immediately imaged and the collection of all the images was finished within 1 hour after dissection. The other half of each specimen was used for histological analysis.

To investigate the optimum conditions for the nonlinear optical imaging of the pancreatic tissues, the nonlinear optical images of cold stored, fixed, and cryopreserved rat pancreatic tissues were compared with those of the fresh tissues respectively, since the structure of the tissues can be maintained by fixation and it has been reported that the intrinsic fluorescent components (NAD(P)H and FAD) can be detected with a high degree of accuracy below −80°C [23]. The cold stored pancreatic tissues were stored in 4°C for 4 hours, 12 hours, and 24 hours after excision, respectively. The fixed tissues were processed with 4% paraformaldehyde in PBS for 19 hours. The cryopreserved tissues were frozen to −196°C in liquid nitrogen for at least 30 minutes. All the tissues were transferred to PBS after processing and then imaged at room temperature.

### Nonlinear optical imaging

**Figure 1:**
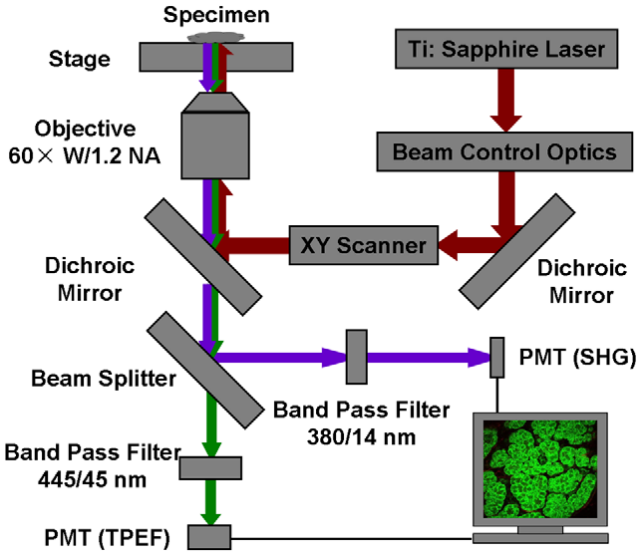
Schematic of a nonlinear optical microscopic system.

Nonlinear optical imaging was performed using a modified system based on a commercial microscope (Fluoview 1000, Olympus, Japan) equipped with a Ti:Sapphire laser (Mai Tai, Spectra-Physics) with a repetition rate of 80 MHz (Fig. 1). The 750-nm output of the laser system was used for excitation except where otherwise noted. The average laser power on the surface of the sample was about 15 mW. The scanning rate is 10 µs/pixel. The emitted signals are collected by the focusing objective (60×/1.2 NA water-immersion, Olympus). Two band-pass filters (FF01-380/14-25 and FF01-445/45-25, Semrock) were employed before the photomultiplier tubes to detect the SHG and TPEF signals, respectively. The SHG signal was confirmed by the property of wavelength dependence, since there was no signal detected in the 380/14 nm range at wavelengths longer than 780 nm. The 3D volume rendering of ImageJ (National Institute of Health, Bethesda, Maryland) was used for image presentation.

### Quantitative analysis of nuclear size and collagen

For each stage of the subcutaneous pancreatic tumor xenografts, 80 cells in the nonlinear optical images were visually chosen and the diameters of the nucleus were manually measured to estimate nuclear sizes. The average nuclear sizes for the subcutaneous pancreatic tumor xenografts harvested at different stages were calculated, respectively.

For collagen analysis, the content of collagen fibers for each specimen was evaluated by the proportion of the number of pixels that collagen fibers account for to the total number of pixels of the image. 40 axial consecutive images from the surface were calculated to get the average content value for all samples. The gray level co-occurrence matrix (GLCM) method, which is a texture analysis method based on the estimation of the second-order joint conditional probability density function [24], [25], was used to characterize the morphology of collagen fibers.

Statistical significance was calculated with ANOVA linear contrast using SPSS software (SPSS). All p values of 0.05 or less were considered as significant referred to as such in the text.

### Histological Analysis

The pancreatic tissues were fixed with 10% neutral buffered formalin, and paraffin-embedded sections with a thickness of 5 µm were routinely prepared. The tissue sections were subsequently stained with hematoxylin-eosin and Masson's trichrome, respectively. The hematoxylin-eosin stain was used for assessing cellular morphology, and the Masson's trichrome stain was used for the detection of collagen fibers in pancreatic tissues. Histological images were acquired using an optical microscope (IX81, Olympus, Japan) equipped with a 20× air objective and a color digital industrial camera (DFK 41BU02, The Imaging Source Europe GmbH, Germany).

## Results and Discussion

### Visualization of morphological details of the normal rat pancreas

**Figure 2:**
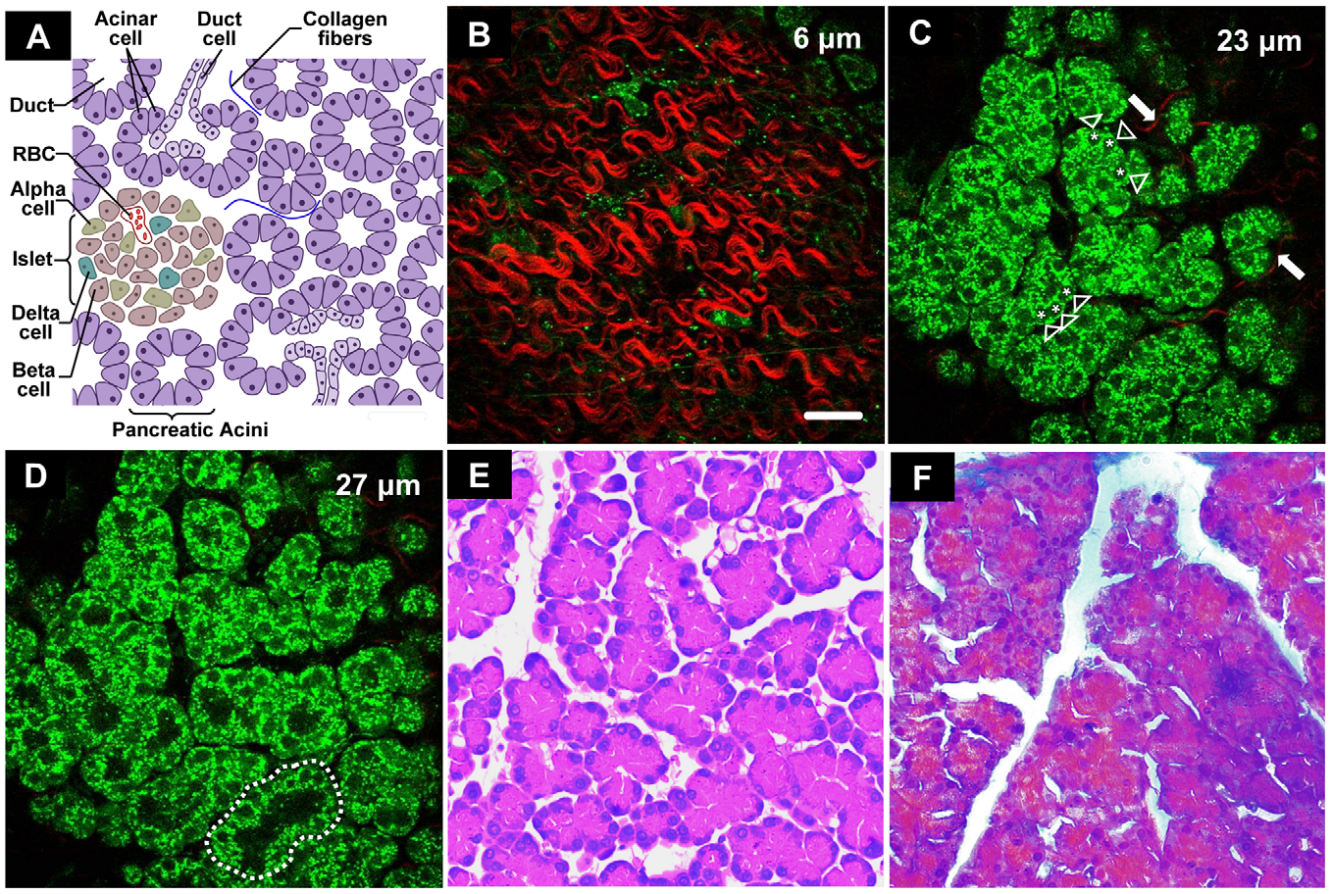
Visualization of the normal rat pancreas using nonlinear optical microscopy and conventional histology. (A) The anatomical organization of the normal rat pancreas is composed of exocrine acini and endocrine pancreatic islets. RBC: red blood cell. (B) The nonlinear optical image of the normal rat pancreas at an imaging depth of 6 µm. The red color-coded structure is collagen, and the green color for fluorescent component. Scale bar is 30 µm. (C) The nonlinear optical image of the normal rat pancreas at an imaging depth of 23 µm. The asterisks, arrowheads and arrows indicate the nuclei, acinar cells, the collagen fibers, respectively. (D) The nonlinear optical image of the normal rat pancreas at an imaging depth of 27 µm. The dotted circle indicates the pattern of pancreatic acini. (E) The pancreatic acini can be observed in the hematoxylin and eosin image. (F) The Masson's trichrome image show small amount of collagen fibers are distributed around the acini of normal rat pancreatic samples.

Pancreas is an important gland with both exocrine and endocrine functions (Fig. 2A) with a thin fibrous connective tissue capsule that enwraps the pancreatic parenchyma. The exocrine portion of the pancreas consists of grape-like clusters of pancreatic acinar cells named acini, which synthesize and secrete digestive enzymes into the duodenum via ductal systems. The pancreatic acinar cells show a pyramidal shape with apical cytoplasm containing zymogen granules and a prominent nucleus located close to the basolateral cell membrane. The endocrine portion of the pancreas accounts for about 1–2% of the total pancreatic mass, and is comprised of scattered pancreatic islets which contain clusters of different kinds of hormone-producing cells.

The nonlinear optical image at an imaging depth of 6 µm shows the fibrous connective tissue capsule in the surface of the pancreas (Fig. 2B), as indicated by the SHG signal. In the pancreatic parenchyma, the nonfluorescent cell nuclei appear as dark and round regions located close to the basolateral cell membrane (asterisks in Fig. 2C) and the individual pancreatic acinar (arrowheads in Fig. 2C) cells can be clearly identified in superficial layer of the acini (imaging depth of 23 µm). Besides, a small amount of collagen fibers (arrows in Fig. 2C) distributed in the extracellular matrix around the acini, which is in correspondence with Masson's trichrome-stained images (Fig. 2F). Figure 2D shows that the acinar cells are arranged in a pattern (dotted circle) similar to the structural assembly of the exocrine acini (Fig. 2A) at the deep layer (imaging depth of 27 µm). The grape-like clusters of the acinar cells can also be observed in the corresponding hematoxylin-eosin-stained microscopic images (Fig. 2E). However, compared with the nonlinear optical microscopic images, the histological images are hard to visualize the fibrous capsule and the 3-D conformation of the acinar cells. A supplemental movie is also provided to show a depth-resolved stack of the pancreas (Video S1). Besides, the 3-D arrangement of the acinar cells is further validated by staining the nuclei with a fluorescent dye (Figure S1). Our results demonstrate that the nonlinear microscopy has the advantage of 3-D visualization of the pancreas without tissue slicing or co-registration over the histological methods.

The normal pancreatic islet is reported to be pervaded by a dense network of capillaries and surrounded by a thin collagen capsule and glial sheet that separates the endocrine cells from the exocrine component [26]. However, there was not any pattern similar to the islets present in the nonlinear optical images, possibly because of the sparse population of islets. Larger area imaging with specific dyes may facilitate further identification of the structure of the pancreatic islets.

The results prove that the intrinsic TPEF fluorescence of the pancreatic cells and the SHG signal of the collagen fibers in the extracellular matrix can visualize the morphology of the pancreatic cells and extracellular components by using nonlinear optical microscopy.

### Origin of intrinsic contrast

It is well documented in the literature that the major sources of TPEF signal in the cytoplasm are NAD(P)H and FAD with emission spectra that peak at 460 nm and 530 nm, respectively [13], [14], [27]. We detected the autofluorescence in the range of 500–550 nm at the excitation wavelength of 750 nm and found that the intensity is much lower than that in the range of 425–470 nm (data not shown). Since the intrinsic fluorophores show strong emission in the 425–470 nm channel when excited at 750 nm, we speculate that the intrinsic fluorescence of the pancreatic tissues mainly comes from NAD(P)H, and FAD has a minor contribution to the intrinsic emission.

Fibrillar collagen has been reported to show a non-centrosymmetrical structure, thus making it a primary contributor to the SHG signal in the biological tissues [13], [28]. According to the morphology and distribution of the fibers revealed by the SHG images, the origin of the SHG signal from the pancreas is expected to be the collagen fibers in the extracellular matrix.

### Optimum conditions for intrinsic imaging of pancreatic tissues

**Figure 3:**
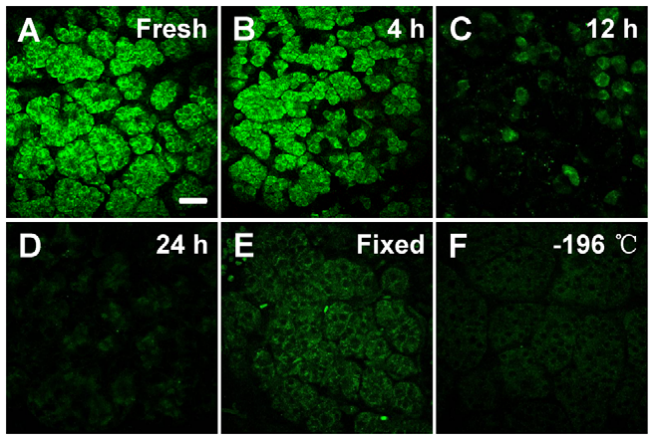
The TPEF images of the pancreatic samples prepared under different conditions. The intrinsic fluorescence of (A) the fresh tissues, tissues stored in 4°C for (B) 4 hours, (C) 12 hours, and (D) 24 hours, (E) fixed tissues as well as (F) frozen tissues were detected. Scale bar is 30 µm.

It has been reported that the concentration of NAD(P)H and FAD is associated with the redox ratio, which is sensitive to changes in the cellular metabolic rate and vascular oxygen supply [29]. Therefore, we have imaged 4°C stored, fixed, and cyropreserved pancreatic tissues to investigate the optimum conditions for the nonlinear optical imaging of the pancreatic tissues. Compared with the fresh tissues (Fig. 3A), the nonlinear optical images of the pancreatic tissues stored in 4°C for 4 hours show the morphology of the pancreatic acini with lower contrast (Fig. 3B). Only a few pancreatic cells can be observed with decreased intensity of fluorescence in the pancreatic tissue stored in 4°C for 12 hours (Fig. 3C)), while the intrinsic fluorescence become negligible and the cells can be hardly identified for those stored in 4°C for 24 hours (Fig. 3D). The reduction of the intensity of the intrinsic fluorescence and the loss of the pancreatic cells may result from the decrease of viability of the pancreatic tissues under the deprivation of oxygen and nutrition after resection. Particularly, the process of autodigestion induced by the injury of the pancreas may probably accelerate the damage of the pancreatic tissues.

Figure 3E shows that the organization of the intact pancreatic acini was preserved after fixation. However, the intensity of the fluorescence decreased significantly, possibly due to protein denaturation during the process of fixation and the changes in quencher concentrations [29]. In comparison, the cryopreserved pancreatic tissues showed even lower fluorescence in Figure 3F, since the cellular NAD(P)H may vanish because of the death of the cells after the freeze and thawing to room temperature during imaging.

The above results show that the morphology of the pancreatic tissues can be most accurately delineated by imaging fresh tissues without additional processing. Therefore, the fresh pancreatic tissues incubated in PBS were used for subsequent analysis. We have also investigated the optimum excitation wavelength for the nonlinear optical imaging of the pancreatic tissues, since the intrinsic fluorescence is relatively weak. The excitation wavelength is tuned in the 730–840 nm range to perform pancreatic tissue imaging. It was found that 750 nm was the optimal wavelength for the microscopic imaging system we used. The laser power on the surface of the samples was tested to achieve images with high signal to noise ratio. In the case of an average power of about 15 mW, there was no visible photodamage or photobleaching after the acquisition of a series of consecutive axial slices.

### Comparison of normal and cancerous pancreatic tissues

**Figure 4:**
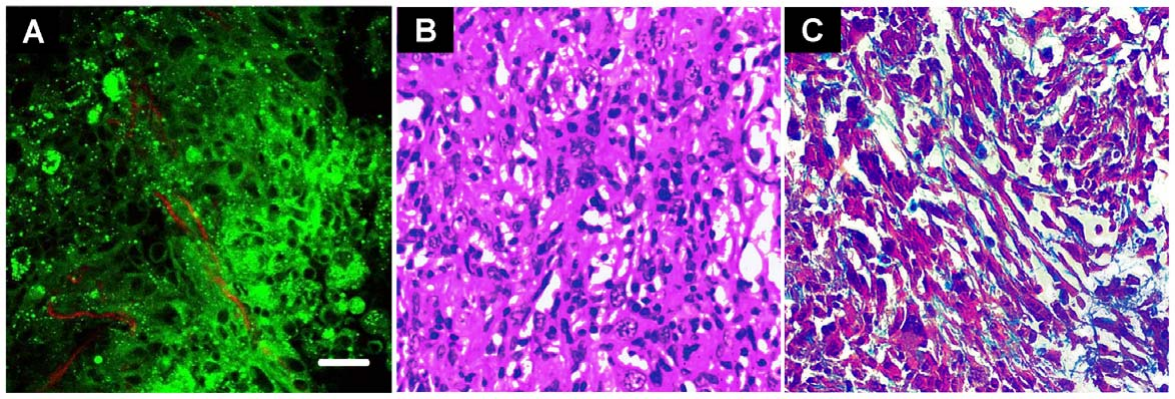
Visualization of the cancerous rat pancreatic samples using nonlinear optical microscopy and conventional histology. (A) The pancreatic cancer cells with various size and shape as well as linear collagen fibers can be identified in the nonlinear optical image. The red color-coded structure is collagen and the green color for fluorescent component. Scale bar is 30 µm. (B) The hematoxylin and eosin image and (C) the Masson's trichrome image show the morphology of cancerous pancreatic tissues in correspondence with (A).

The chemical-induced pancreatic cancer tissues were imaged and associated with the histological images (Fig. 4). Compared with the normal pancreas, larger nuclear size and marked variation in nuclear size and shape can be observed in the chemical-induced pancreatic cancer tissues, which is coincident with the typical characteristics of cancer cells. Besides, the density of the collagen fibers increased, which resembles the intensive stromal fibrosis manifested in pancreatic cancer patients [30]. The obvious differences of the morphological appearance between the normal and the chemical-induced pancreatic cancer tissues indicate that the nonlinear optical methods show the ability to detect neoplastic lesions in abnormal pancreatic tissues.

### Growth of the subcutaneous pancreatic tumor xenografts

**Figure 5:**
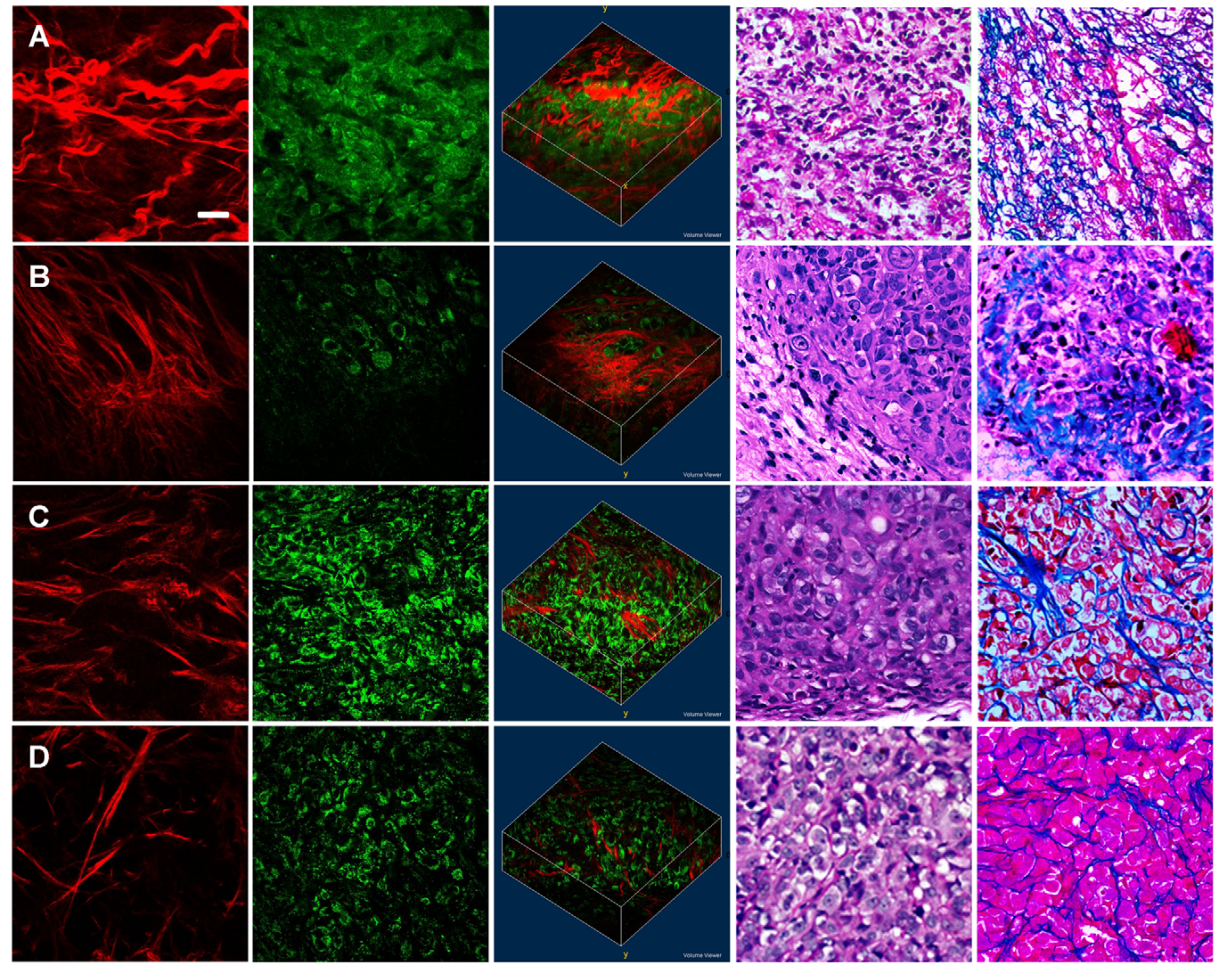
The nonlinear optical images and corresponding histology of subcutaneous pancreatic tumor xenografts. The pancreatic tumor xenografts harvested at different stages, including (A) 5 days, (B) 10 days, (C) 20 days, and (D) 30 days after implantation, were imaged and related with conventional histology. The SHG images (red color-coded), the TPEF images (green color-coded), and the 3-D superimposed SHG/TPEF images are shown in the first three columns respectively, while the hematoxylin and eosin images and the Masson's trichrome images are displayed in the last two columns. All the 3-D images are 211 µm×211 µm×50 µm. Scale bar is 30 µm.

The subcutaneous pancreatic tumor xenografts harvested at 5 days after inoculation mainly consists of wavy collagen fibers and small cells which may be leucocytes (Fig. 5A). In comparison, the cells in the tumor xenografts harvested at 10 days after inoculation show enlarged nuclei, indicating that the cancer cells begin to proliferate (Fig. 5B). Besides, the collagen fibers are linear and arranged radiant from the cancer cells, which may contribute to tumor pervasion along the fibers. The amounts of tumor cells increased in the tumor tissues harvested at 20 days after inoculation (Fig. 5C), while it decreased in those harvested at 30 days after inoculation (Fig. 5D), implying that there is necrosis in the center of the tumor. As the xenografts develop, the collagen fibers get more fragmented and irregular, and the density of the fibers decreased, which may be associated with the degrading and remodeling of the extracellular matrix during the growth of the tumor xenografts.

The histological images show that extensive necrosis is present in the tumor xenografts harvested at 5 days after inoculation, and most of the cells are neutrophils, which contain three or four nuclear lobes (Fig. 5A). At 10 days after implantation, most of the cells are tumor cells, and abundant long linear collagen fibers are present (Fig. 5B). At 20 days after implantation, high density of tumor cells can be seen and relatively lower content of collagen fibers occurs (Fig. 5C). Finally, at 30 days after implantation, scattered necrosis can be observed in the central of the tumor (Fig. 5D). We found that the results of label-free TPEF and SHG imaging are consistent with the traditional histological staining methods.

**Figure 6:**
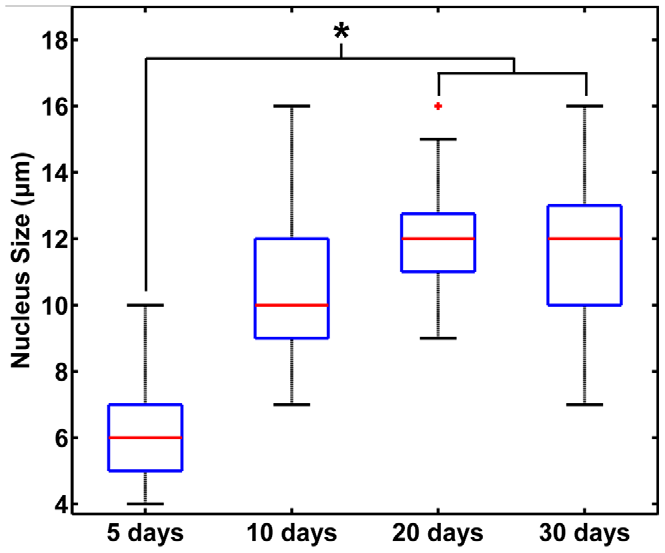
The nuclear sizes for the subcutaneous pancreatic tumor xenografts harvested at different stages. The nuclear size at 5 days is significantly lower than those at 20 days and 30 days. *, p<0.001, ANOVA linear contrast. The sample size is 5 for each group (5 mice). +, outliers on the box-and-whisker diagram; *bars*, total extent of the data.

**Figure 7:**
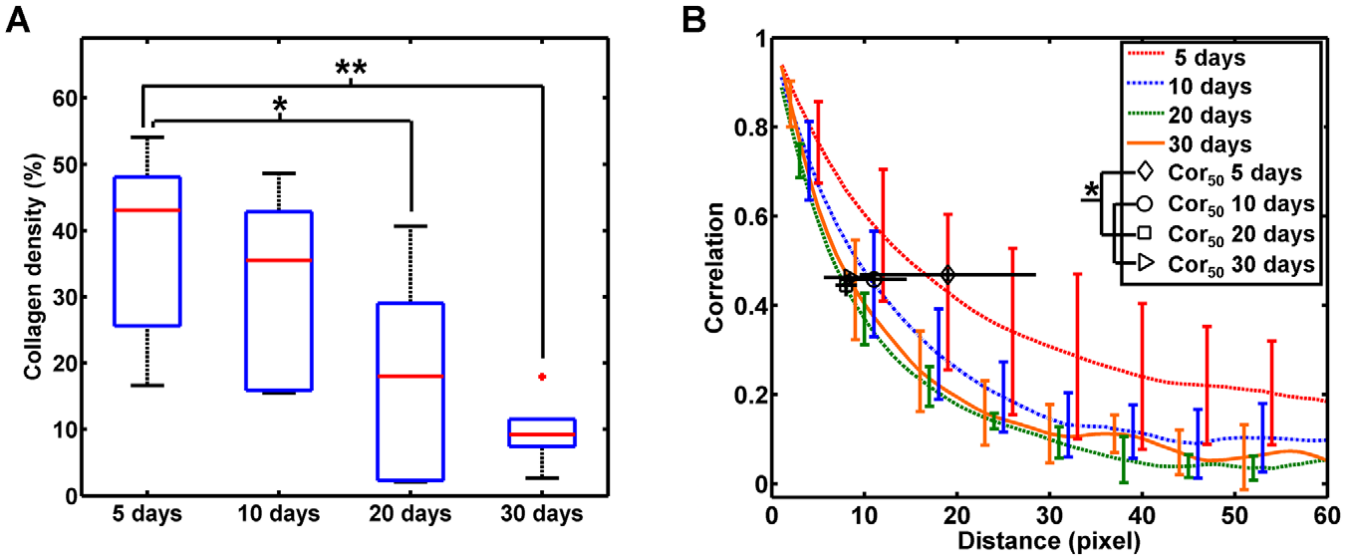
Quantitative analysis of the collagen fibers in the subcutaneous pancreatic tumor xenografts. (A) The collagen density decreases as the tumor xenografts grow. The density of the tumors at 5 days is significantly higher in comparison to those at 20 days (*, p = 0.033, ANOVA linear contrast) and 30 days (**, p = 0.005, ANOVA linear contrast). +, outliers on the box-and-whisker diagram; *bars*, total extent of the data. (B) The organization of collagen fibers changes during the growth of the subcutaneous pancreatic tumor xenografts. An overall comparison of the correlation values shows the greatest difference between the tumor xenografts harvested at 5 days and those harvested after 10 days, as indicated by the Corr_50_ value, the distance where the correlation crossed 50% of the initial correlation. *, p = 0.035, ANOVA linear contrast. The sample size is 5 for each group (5 mice). Error bars are one standard deviation above and below each data point.

Quantitative analysis of the nuclear size shows that the diameter of the cell nuclei harvested at 10 days after inoculation falls between that harvested at 5 days (about 6 µm) and those harvested after 20 days (mainly ranged between 10 µm and 13 µm). This indicates that the proporation of tumor cells to neutrophils increases as the xenografts grow (Fig. 6, p<0.001, ANOVA linear contrast between the size at 5 days and those after 20 days; n = 5 for each group). It reveals that the nuclear size of the cells can be obtained using nonlinear optical imaging, which is important for the detection of tumor cells. The density of the collagen fibers has also been calculated for the tumor xenografts harvested at different stages respectively. It can be seen that the amount of the collagen fibers gradually decreased as the tumor grows and the density at 5 days differed significantly from those after 20 days (Fig. 7A). For the quantification of the structural changes of the collagen fibers, the GLCM method was used for texture analysis. Correlation is a textural feature extracted from GLCM and can be used to estimate the direction of the texture. As Figure 7B indicates, the correlation curves of the tumor tissues harvested after 10 days are lower than those harvested at 5 days, corresponding to more disordered collagen fibers. The Corr_50_, calculated by the pixel distance where the correlation dropped to 50% of the initial value, is significantly greater in the xenografts harvested at 5 days compared with those harvested at 10 days, 20 days, and 30 days (Fig. 7B; p = 0.035, ANOVA linear contrast; n = 5 for each group). The above quantitative results are consistent with the visual appearance of the nonlinear optical images.

The neutrophils and necrosis observed in the subcutaneous tumor xenografts harvested at 5 days are possibly due to the inflammatory response during the tumor-host reaction, since it has been reported that the residual immune system of the host may participate in tumor regression in the early stages after the inoculation of tumor xenografts [31]. The density of cancer cells increased from 10 days to 20 days, while it decreased and scattered necrosis appeared at 30 days. The necrosis in the subcutaneous tumor xenografts harvested at 30 days results from a poor vasculature [31].

The above results show that the process of the growth of the subcutaneous tumor xenografts can be visualized by the nonlinear optical microscopy, suggesting that the nonlinear optical methods might aid in the evaluation of the severity or staging of the pancreatic cancer. However, the growth of the subcutaneous tumor xenografts is not equal to the natural process of the development of the pancreatic cancer [14], [15]. More in-depth studies on the spontaneous pancreatic cancer model such as the chemical-induced pancreatic cancer are necessary to validate the ability of nonlinear optical methods in the early detection of this disease. Further applications to the genetically manipulated mouse model that recapitulates the human disease and the human pancreatic tissues obtained during the surgical resection may provide more information for the detection of abnormal lesions and potential clinical translation. It might also be valuable for potential intravital imaging in the future when suitable intravital imaging instruments are developed.

## Supporting Information

Video S1 **Axial superimposed TPEF/SHG stack of the normal rat pancreas.** This movie displays the 3-D morphology of the normal rat pancreas spanning from the fibrous surface to the acini with a depth of 29 µm. The TPEF and SHG signals are green and red color-coded, respectively. Scale bar is 30 µm.
(AVI)

Figure S1 **Single-photon 3-D images of the normal acini stained by Hoechst 33342.** The normal pancreatic tissues are stained with fluorescent dye Hoechst 33342 (10 µg/ml). Images were acquired at an excitation wavelength of 405 nm, at various depths within the tissues as indicated by the z value in the upper right corner of each image. The arrangement of pancreatic acinar cells can be clearly seen, showing the 3-D structure of the acini (dotted circle). Scale bar is 30 µm.
(TIF)
