# Supplementary figures and images for: Nonlinear Optical Microscopy for Histology of Fresh Normal and Cancerous Pancreatic Tissues
Source: PLoS One. 2012 May 24;7(5):e37962. doi: 10.1371/journal.pone.0037962 (PMC3360059; doi:10.1371/journal.pone.0037962)

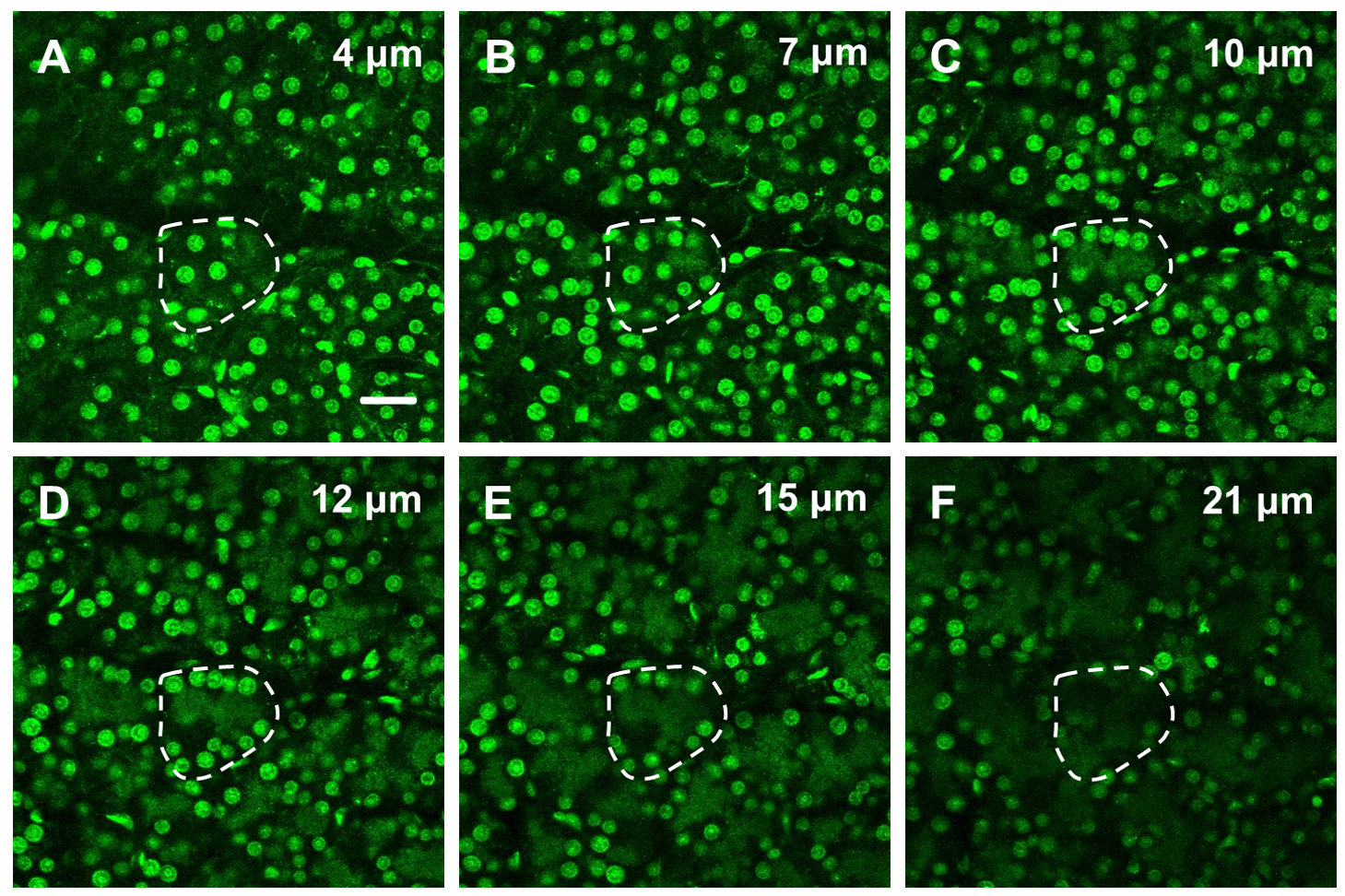

Supplement: Figure S1 — Single-photon 3-D images of the normal acini stained by Hoechst 33342. The normal pancreatic tissues are stained with fluorescent dye Hoechst 33342 (10 µg/ml). Images were acquired at an excitation wavelength of 405 nm, at various depths within the tissues as indicated by the z value in the upper right corner of each image. The arrangement of pancreatic acinar cells can be clearly seen, showing the 3-D structure of the acini (dotted circle). Scale bar is 30 µm. (TIF) [file pone.0037962.s002.tif]
